# Supplementary material for: Dipper throated optimization with deep convolutional neural network-based crop classification for remote sensing image analysis
Source: PeerJ Comput Sci. 2024 Jan 25;10:e1828. doi: 10.7717/peerj-cs.1828 (PMC10909238; doi:10.7717/peerj-cs.1828)
Supplement: Supplemental Information 1 [file peerj-cs-10-1828-s001.docx]

import java.awt.image.BufferedImage;

import java.io.File;

import java.io.IOException;

import java.util.Arrays;

import java.util.Random;

import javax.imageio.ImageIO;

import org.apache.commons.math3.ml.clustering.DoublePoint;

import org.apache.commons.math3.ml.clustering.SphericalKMeansClusterer;

import org.deeplearning4j.nn.api.OptimizationAlgorithm;

import org.deeplearning4j.nn.conf.layers.DenseLayer;

import org.deeplearning4j.nn.conf.MultiLayerConfiguration;

import org.deeplearning4j.nn.conf.NeuralNetConfiguration;

import org.deeplearning4j.nn.conf.inputs.InputType;

import org.deeplearning4j.nn.multilayer.MultiLayerNetwork;

import org.deeplearning4j.nn.weights.WeightInit;

import org.deeplearning4j.optimize.listeners.ScoreIterationListener;

import org.nd4j.linalg.activations.Activation;

import org.nd4j.linalg.api.ndarray.INDArray;

import org.nd4j.linalg.dataset.DataSet;

import org.nd4j.linalg.dataset.api.iterator.BaseDatasetIterator;

import org.nd4j.linalg.factory.Nd4j;

import org.nd4j.linalg.lossfunctions.LossFunctions;

public class CropClassification {

private static int numLabels = 6; // Assuming 6 crop types

private static int seed = 123;

private static int iterations = 1000;

private static int batchSize = 64;

public static void main(String[] args) throws IOException {

**Load images and corresponding labels**

BufferedImage[] images = loadImages("path_to_image_folder");

int[] labels = loadLabels("path_to_label_file");

**Preprocess images and convert them to features**

double[][] features = extractFeatures(images);

**Cluster features into clusters**

int numClusters = 50; // Number of clusters based on your choice

double[][] clusteredFeatures = kMeansClustering(features, numClusters);

**Create a new set of images based on the clustered features**

BufferedImage[] newImages = createNewImages(clusteredFeatures);

**Preprocess the new images and convert them to features**

double[][] newFeatures = extractFeatures(newImages);

**Create DataSet**

DataSet dataSet = createDataSet(newFeatures, labels);

**Create the neural network model**

MultiLayerNetwork model = createModel();

**Train the model**

model.fit(dataSet);

**Evaluate the model**

Evaluation eval = new Evaluation(numLabels);

INDArray output = model.output(dataSet.getFeatures());

eval.eval(dataSet.getLabels(), output);

System.out.println(eval.stats()); }

private static BufferedImage[] loadImages(String path) throws IOException

{**Implement this method to load images from the specified path**}

private static int[] loadLabels(String path) throws IOException {

**Implement this method to load labels from the specified path** }

private static double[][] extractFeatures(BufferedImage[] images) {

**Implement this method to extract features from the images using the GoogleNet Model** }

private static double[][] kMeansClustering(double[][] features, int numClusters) {

SphericalKMeansClusterer<DoublePoint> clusterer = new SphericalKMeansClusterer<>(numClusters);

DoublePoint[] dataPoints = new DoublePoint[features.length];

for (int i = 0; i < features.length; i++) {

dataPoints[i] = new DoublePoint(features[i]);

}

return Arrays.stream(clusterer.cluster(dataPoints)).map(DoublePoint::getPoint).toArray(double[][]::new);

}

private static BufferedImage[] createNewImages(double[][] clusteredFeatures) {

**Implement this method to create new images based on the clustered feature** }

private static DataSet createDataSet(double[][] newFeatures, int[] labels) {

int numSamples = newFeatures.length;

INDArray features = Nd4j.create(newFeatures);

INDArray labelsArr = Nd4j.create(numSamples, numLabels);

for (int i = 0; i < numSamples; i++) {

labelsArr.putScalar(i, labels[i], 1.0);

}

return new DataSet(features, labelsArr);

}

private static MultiLayerNetwork createModel() {

MultiLayerConfiguration conf = new NeuralNetConfiguration.Builder()

.seed(seed)

.weightInit(WeightInit.XAVIER)

.updater(new Nesterovs(0.001, 0.9))

.list()

.layer(0, new DenseLayer.Builder().nIn(256 * 6 * 6).nOut(1024).activation(Activation.RELU).build())

.layer(1, new DenseLayer.Builder().nIn(1024).nOut(1024).activation(Activation.RELU).build())

.layer(2, new DenseLayer.Builder().nIn(1024).nOut(numLabels).activation(Activation.SOFTMAX).build())

.backprop(true).pretrain(false)

.build();

MultiLayerNetwork model = new MultiLayerNetwork(conf);

model.init();

model.setListeners(new ScoreIterationListener(10));

return model;

}

}

**Existing Algorithm comparison with proposed algorithm**

import java.awt.Color;

import java.awt.image.BufferedImage;

import java.io.File;

import java.io.IOException;

import java.util.HashMap;

import java.util.Map;

import javax.imageio.ImageIO;

import org.deeplearning4j.nn.api.OptimizationAlgorithm;

import org.deeplearning4j.nn.conf.layers.DenseLayer;

import org.deeplearning4j.nn.conf.layers.OutputLayer;

import org.deeplearning4j.nn.conf.layers.SubsamplingLayer;

import org.deeplearning4j.nn.conf.NeuralNetConfiguration;

import org.deeplearning4j.nn.conf.inputs.InputType;

import org.deeplearning4j.nn.weights.WeightInit;

import org.deeplearning4j.optimize.listeners.ScoreIterationListener;

import org.nd4j.linalg.activations.Activation;

import org.nd4j.linalg.dataset.DataSet;

import org.nd4j.linalg.lossfunctions.LossFunctions;

public class CropClassification {

private static final int NUM_OUTPUTS = 4; // Number of output classes

private static final int[] IMAGE_SIZE = {24, 24, 3}; // Height x Width x Channel

private static final int BATCH_SIZE = 64;

private static final int ITERATIONS = 1000;

private static final double LEARNING_RATE = 0.001;

public static void main(String[] args) throws IOException {

**Load your training dataset and corresponding labels**

DataSet dataSet = loadDataSet("path/to/training/images", "path/to/training/labels");

**Create a CNN model**

MultiLayerConfiguration conf = new NeuralNetConfiguration.Builder()

.seed(123)

.optimizationAlgo(OptimizationAlgorithm.STOCHASTIC_GRADIENT_DESCENT)

.iterations(ITERATIONS)

.learningRate(LEARNING_RATE)

.weightInit(WeightInit.XAVIER)

.updater(new Nesterovs(LEARNING_RATE, 0.98))

.list()

.layer(0, new ConvolutionLayer.Builder(5, 5)

.nIn(3)

.stride(1, 1)

.nOut(20)

.activation(Activation.IDENTITY)

.build())

.layer(1, new SubsamplingLayer.Builder(SubsamplingLayer.PoolingType.MAX)

.kernelSize(2, 2)

.stride(2, 2)

.build())

.layer(2, new ConvolutionLayer.Builder(5, 5)

.nIn(20)

.stride(1, 1)

.nOut(50)

.activation(Activation.IDENTITY)

.build())

.layer(3, new SubsamplingLayer.Builder(SubsamplingLayer.PoolingType.MAX)

.kernelSize(2, 2)

.stride(2, 2)

.build())

.layer(4, new DenseLayer.Builder().activation(Activation.RELU)

.nIn(50 * 6 * 6)

.nOut(500)

.build())

.layer(5, new OutputLayer.Builder(LossFunctions.LossFunction.NEGATIVELOGLIKELIHOOD)

.nIn(500)

.nOut(NUM_OUTPUTS)

.activation(Activation.SOFTMAX)

.build())

.backprop(true).pretrain(false)

.build();

MultiLayerNetwork model = new MultiLayerNetwork(conf);

model.init();

**Setup a listener to score the model every iteration**

model.setListeners(new ScoreIterationListener(ITERATIONS / 10));

**Train the model**

model.fit(dataSet);

**Classify the crops of a new image**

File newImageFile = new File("path/to/new/image");

BufferedImage newImage = ImageIO.read(newImageFile);

Map<BufferedImage, Integer> classifiedCrops = classifyCrops(newImage, model);

**Based on the majority vote of the classified crops, classify the entire image**

int predictedClass = majorityVote(classifiedCrops.values());

System.out.println("Predicted class for the entire image: " + predictedClass);

}

**Implement this method to load your dataset**

private static DataSet loadDataSet(String imagesPath, String labelsPath) {

throw new UnsupportedOperationException("Not implemented yet");

}

**Implement this method to extract features from an image**

private static INDArray extractFeatures(BufferedImage image, MultiLayerNetwork model) {

throw new UnsupportedOperationException("Not implemented yet");

}

**Classify the crops of an image**

private static Map<BufferedImage, Integer> classifyCrops(BufferedImage image, MultiLayerNetwork model) {

Map<BufferedImage, Integer> classifiedCrops = new HashMap<>();

// Crop the image and extract features from each crop

for (BufferedImage crop : getCrops(image)) {

INDArray features = extractFeatures(crop, model);

// Classify the crop

int predictedClass = model.output(features).argMax(1).getInt(0);

classifiedCrops.put(crop, predictedClass);

}

return classifiedCrops;

}

**Implement this method to get the crops of an image**

private static List<BufferedImage> getCrops(BufferedImage image) {

throw new UnsupportedOperationException("Not implemented yet");

}

**Determine the majority vote of the classified crops**

private static int majorityVote(Collection<Integer> votes) {

int[] countByClass = new int[NUM_OUTPUTS];

for (int vote : votes) {

countByClass[vote]++;

}

return IntStream.range(0, NUM_OUTPUTS).reduce(0, (a, b) -> countByClass[a] > countByClass[b] ? a : b);

}

}
